# Feasibility of LifeLab Dublin: a co-designed health literacy intervention for socioeconomic disadvantaged schools

**DOI:** 10.1093/heapro/daag127

**Published:** 2026-08-18

**Authors:** Caera L Grady, Lorna Burke, Sarahjane Belton, Maeve Murray, Cliona Power, Hannah R Goss

**Affiliations:** School of Health and Human Performance, Faculty of Science and Health, Dublin City University, Dublin D09 YH9P, Ireland; School of Health and Human Performance, Faculty of Science and Health, Dublin City University, Dublin D09 YH9P, Ireland; School of Health and Human Performance, Faculty of Science and Health, Dublin City University, Dublin D09 YH9P, Ireland; School of Health and Human Performance, Faculty of Science and Health, Dublin City University, Dublin D09 YH9P, Ireland; School of Health and Human Performance, Faculty of Science and Health, Dublin City University, Dublin D09 YH9P, Ireland; School of Health and Human Performance, Faculty of Science and Health, Dublin City University, Dublin D09 YH9P, Ireland

**Keywords:** health literacy, adolescents, secondary school, programme, acceptability

## Abstract

High health literacy (HL) is associated with improved health behaviours and outcomes. Schools are an ideal environment to improve adolescents’ HL levels. This study assessed the feasibility of LifeLab Dublin, a co-designed HL intervention, for socioeconomically disadvantaged schools. Students (*N* = 356) and teachers (*N* = 15) from four Dublin schools participated in this study. A pre–post follow-up, single-arm feasibility, mixed methods study design was adopted, guided by the Orsmond and Cohn (2015) feasibility objectives. Data sources included research records, semistructured interviews and focus groups, and student and teacher surveys. A combined inductive–deductive reflexive thematic analysis was conducted on the interview and focus group data. Descriptive statistics were run on survey data. Student surveys measured HL, wellbeing, and health-related quality of life at baseline, postintervention, and follow-up. Data sources were analysed independently and integrated during write up. Interviews and focus group data indicated positive experiences with the LifeLab programme among students and teachers. School-level recruitment rates were low (6% participated), but the individual level was strong (91% participated). Attrition rates were 13.8% postintervention and 16.8% at follow-up. Including additional content for each health topic, flexibility in delivery, and the inclusion of structured teacher training may improve the programme. Moderate HL levels and good wellbeing was observed at baseline. Mean increases were observed from baseline to follow-up in students’ HL, wellbeing, and health-related quality of life. Evidence supports the feasibility and acceptability of LifeLab for young adolescents and teachers, with progression to a definitive trial as a logical next step.

Contribution to health promotion

- This study outlines a feasible school-based health literacy (HL) intervention for adolescents from socioeconomic disadvantaged backgrounds that is acceptable for students and teachers.
- This study shows evidence of promise that LifeLab will improve adolescents’ HL, wellbeing, and health-related quality of life.
- This study recommends future school-based HL interventions with adolescents consider flexibility in intervention delivery, alignment with school curriculum to increase uptake, identifying essential and desirable intervention criteria, and embedding teacher training into the programme.

## Background

Adolescence is a distinct period of rapid growth and development in which changes occur physically, socially, emotionally, and psychologically (World Health Organisation 2017, Sawyer *et al*. 2018). Adolescent health and wellbeing are a worthwhile investment for public health today, the future ageing population, and the next generation of children (Patton *et al*. 2016). There are many factors that can influence adolescents’ adoption or uptake of positive or negative health behaviours such as peer influence and socioeconomic disadvantage (Sawyer *et al.* 2012).

There is global action to improve adolescent health literacy (HL) levels due to its promising impact on health outcomes and role in the prevention of noncommunicable diseases (WHO 2017, Paakkari and Okan 2019). HL is defined as ‘the personal competencies and organizational structures, resources and commitment which enable people to access, understand, appraise and use health information and services in ways which promote and maintain good health’ (Nutbeam and Muscat 2023, pg 3). Education, biology, cognition, and socioeconomic status are all factors that influence adolescents’ lifelong health behaviours (Bay *et al*. 2017). As such, schools have been recognized as a setting which provides an ideal opportunity for young people to develop the necessary health knowledge and skills for life (Tang *et al*. 2009, Pearson *et al*. 2015). Schools have the largest reach over a single population group for such an extended portion of the lifespan regardless of background or socioeconomic status (Story *et al*. 2009). Schools are well-structured educational environments where students develop critical thinking, lifelong learning skills, knowledge, competencies and behaviours (Leger and Nutbeam 2000). Concurrently, school health education curricula are directly linked to improved HL (Nutbeam 2000).

In Ireland, during the 2024/25 academic year there were a total of 232 secondary schools with disadvantaged status, of which 66 were in Dublin (Department of Education and Skills 2025). LifeLab Dublin is a co-designed HL intervention for socioeconomically disadvantaged secondary schools (Goss *et al.* 2021, Goss *et al.* 2022; Smith *et al*. 2022). LifeLab Dublin was developed for ‘Junior Cycle’ students (i.e. the first 3 years of secondary school in Ireland, typically aged 12–15 years) and aligns with the Irish wellbeing curriculum (Smith *et al*. 2022, National Council for Curriculum and Assessment and Department of Education 2023). The Junior Cycle wellbeing curriculum consists of 400 h of timetabled learning, providing opportunities for students to enhance their physical, mental, social, and emotional wellbeing (National Council for Curriculum and Assessment 2021). These hours are divided up between three subject areas: Physical Education, Social, Personal, and Health Education, and Civic, Social and Political Education. LifeLab Dublin was developed in line with the wellbeing curriculum rollout for schools, aiming to support capacity building and buy-in from schools and teachers (Smith *et al*. 2022).

LifeLab Dublin is a 9-week intervention comprising seven classroom lessons and two interactive visits to the Dublin City University (DCU) LifeLab facility. The classroom lessons provide the theoretical foundation for the workshop activities, which are facilitated by undergraduate volunteers under the supervision of the research team. Schools are required to arrange transport to the LifeLab workshops at DCU. Lessons one to three provide an introduction to the LifeLab health topics—such as physical activity, sleep, and nutrition—and engage with LifeLab characters (vignettes) (Goss *et al.* 2021). On Week 4, students attend their first interactive 90-min workshop at DCU focused on exploring factors that impact health. Lessons four to six involve the LifeLab photovoice project where students work in small groups to photograph and discuss health-related factors within their school environment, presenting their findings to the class (Burke *et al.* 2026). On Week 8, the students return to DCU for their second interactive 90-min workshop focused on the impact of lifestyle choices on health. The final lesson allows students to reflect on their learning and set short- and long-term behavioural goals. Further detail on the intervention is provided elsewhere (Goss *et al.* 2021; Smith *et al.* 2024; Burke *et al.* 2026). LifeLab Dublin is part of the LifeLab International Community of Practice, linking with similar but distinct programmes in the UK, Australia and South Africa.

Aligned with phase one of the UK Medical Research Council framework for complex intervention development (Skivington *et al.* 2021), LifeLab Dublin has undergone an iterative and comprehensive intervention development process through co-design with key school actors. The purpose of this study was to assess the feasibility of the LifeLab intervention for students and teachers in socioeconomically disadvantaged schools. The five Orsmond and Cohn (2015) feasibility objectives guided this study; (i) recruitment and resulting characteristics, (ii) appropriateness and suitability of data collection procedures and outcome measures, (iii) suitability and acceptability of study procedures and the intervention, (iv) research team resources and ability to manage study and intervention, and (v) the evidence of promise for the intervention.

## Methods

A 9-week single-arm pre–post follow-up feasibility trial was employed. A convergent mixed methods study design was used, data were collected simultaneously, analysed separately, and merged to address the Orsmond and Cohn (2015) objectives for feasibility studies. The CONSORT statement extension for pilot and feasibility studies was used for reporting this study (Supplementary File S1) (Eldridge *et al*. 2016). Ethical approval was granted from the DCU Research Ethics Committee (No: DCU/REC/2023/152).

### Participants and recruitment

**Figure 1:**
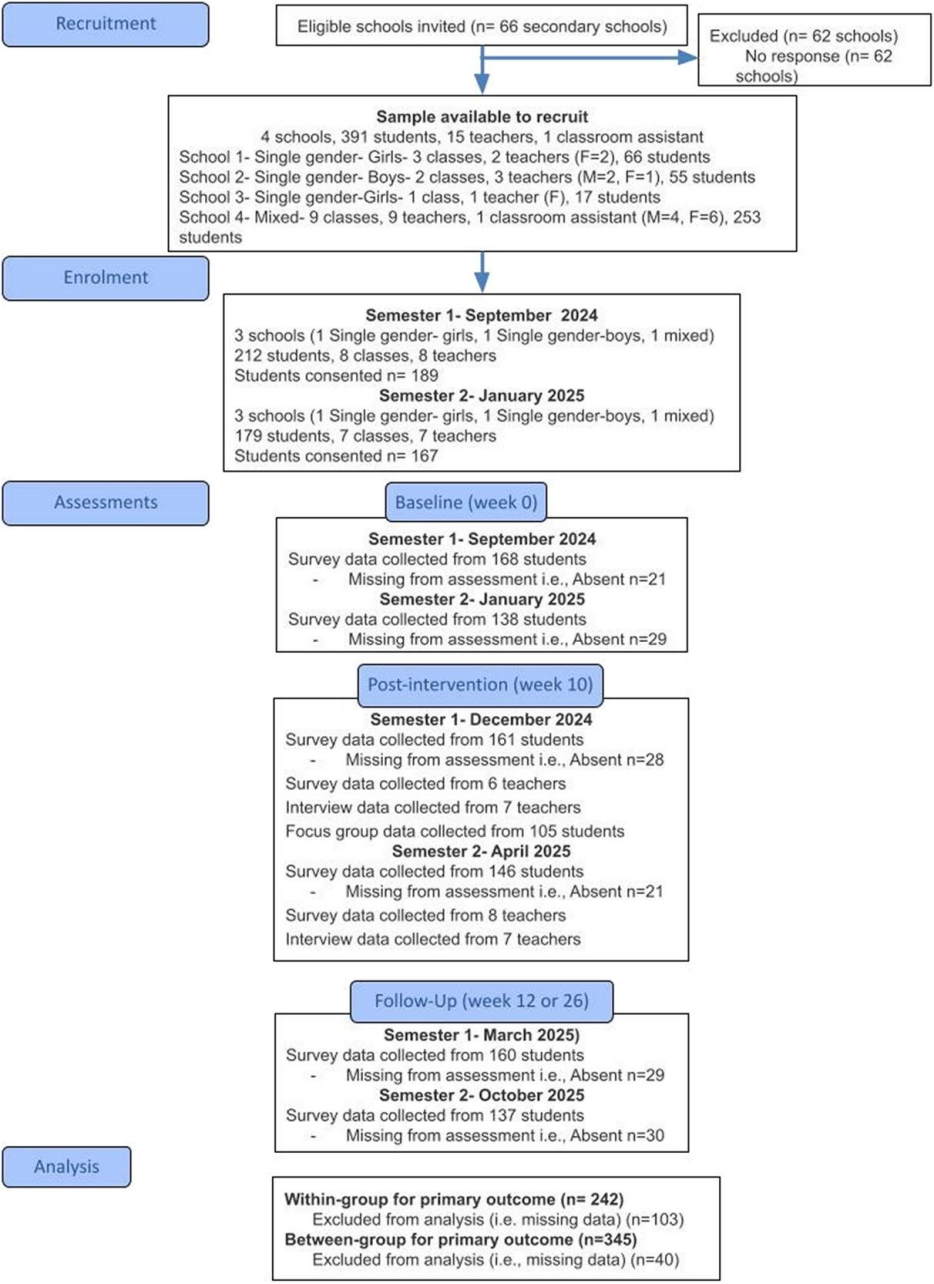
Consort flow diagram of participants included in this study.

Study participants included Junior Cycle students and health education teachers in disadvantaged schools across Dublin. In September 2024, all second level schools with disadvantaged status in Dublin were invited via email to participate in the LifeLab intervention (*N* = 66). Individual phone calls were made to the secretary of each school to further encourage a response to the initial invitation. Thereafter, the research team harnessed existing connections within these schools to encourage uptake. The senior leadership team were asked to recruit health education teacher(s) to deliver the intervention during their timetabled health education class and participate in both surveys and semistructured interviews. The school leaders were then asked to recruit classes of students in Junior Cycle (∼12–15 years) to participate in the LifeLab programme, surveys, and focus groups. A total of four schools agreed to participate in this study between September 2024 and September 2025 (Fig. 1). Purposive sampling was used to recruit students to participate in focus groups. The semester one cohort was invited to participate to ensure the research team could manage the data collection timelines efficiently with the available capacity.

### Procedure

Prior to the intervention, written consent and assent were obtained from all schools, participants, and parents or guardians following the distribution of plain language statements. The gatekeeper (i.e. senior leadership team) provided consent for the school to participate in LifeLab. The class teacher provided consent for their participation in the study and to deliver the LifeLab programme within their classroom. Parents or guardians provided consent for their child to participate in the study. The student provided their assent to participate in the study. Participants, and their parents or guardians, were informed that they could withdraw participation at any time up until the anonymization of the data at the end of the study. If a student did not want to participate in the study they could still participate in the LifeLab programme as it was part of the normal classroom curriculum.

Data were collected over a period of 13 months with two enrolment periods to maximize participation as outlined in Fig. 1.

**Figure 2:**
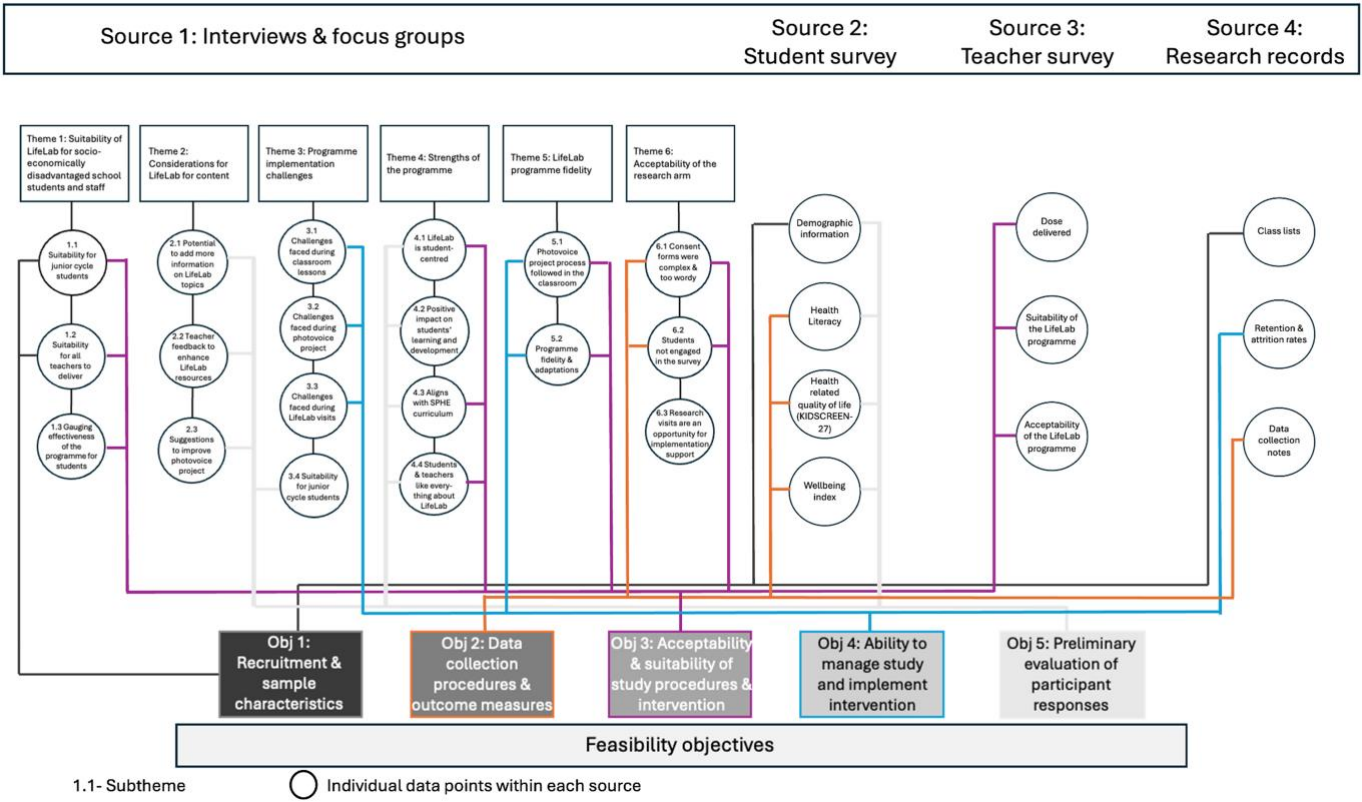
Mapping of the various data and data sources to Orsmond and Cohn (2015) feasibility study objectives.

The Orsmond and Cohn (2015) objectives for feasibility studies were assessed by collecting four different sources of data including, interviews and focus groups, student surveys, teacher surveys, and researcher records (Fig. 2).

Interview and focus group guides (Supplementary File S2) were designed to explore the feasibility, acceptability, and suitability of LifeLab for teachers and young adolescents in disadvantaged schools. Postintervention in the first semester, all schools were invited to participate in a focus group to discuss the students’ experience with LifeLab. Each focus group was facilitated by one member of the research team (LB, CP, HG, and MM), with six to eight participants, and lasted ∼15 min. The teachers involved in the delivery of LifeLab were invited to participate in an interview in the middle, and at the end of the intervention, interviews lasted on average 15 min. Interviews in the middle of the intervention allowed the researchers to understand the teachers’ perspectives in real-time to avoid recall bias. Interviews and focus groups were audio recorded and later transcribed verbatim removing any identifiable information about the school, students, and teachers before analysis.

Surveys, assessing the preliminary evidence of the intervention, were administered to the students by two members of the research team in each school before, immediately after, and at either 3 (Semester 1 group) or 6 months (Semester 2 group) after the intervention. The follow-up data collection was staggered due to the school's academic calendar and the ability to manage data collection within the research team. Researchers explained the survey purpose and instructions, answered questions or concerns and guided students through the surveys (∼20 min). Teachers participated in a short survey (∼10 min) after the intervention assessing the dose delivered, the feasibility, acceptability, and suitability of the intervention.

### Instruments

The teacher interviews evaluated the LifeLab lesson plans, resources, content, and identified any additional support required. The focus groups assessed students’ experiences with LifeLab. Interview and focus group guides are provided in Supplementary File S2. The teacher survey (Supplementary File S3) assessed the dose delivered, feasibility, suitability, and acceptability of the intervention for teachers, students, and the wellbeing curriculum needs.

Student surveys were used to explore any evidence of promise for LifeLab and consisted of three previously validated measures; HL in School Aged Children (HLSAC) (Paakkari *et al*. 2016), WHO-5 wellbeing index (Sischka *et al*. 2026), and the Kidscreen-27 survey (Ravens-Sieberer *et al*. 2007). The HLSAC survey included 10-items that assessed student knowledge and competencies to promote health from one ‘not at all true’ to four ‘absolutely true’ (Paakkari *et al*. 2016). A sum score was calculated with higher scores indicating higher HL levels. Similarly, a sum score was calculated for the WHO-5 wellbeing index which consists of five reverse-scored items assessing students’ mood on a scale of zero ‘at no time’ to five ‘all of the time’ (Sischka *et al*. 2026). The Kidscreen-27 assessed students’ health-related quality of life with five subscales, related to physical wellbeing, psychological wellbeing, autonomy and parent relations, peer and social support, and school environment (Ravens-Sieberer *et al*. 2007). Sum scores were calculated for each subscale and then replaced with the corresponding Rasch person parameter then finally into T values. Researcher records included note-taking, attendance sheets and class lists to calculate attrition, engagement and response rates from baseline to follow-up assessments.

### Reflexivity and the research team

The first author (CG) joined the research team after the trial and took an objective role in collating, reviewing, analysing, and writing-up the data presented. The remaining authors (LB, MM, CP, SB, and HG) were involved in the study conceptualization, design, recruitment, data collection, reviewing the analysed data, and revising manuscript drafts. Three of the authors are qualified secondary school physical education teachers (LB, MM, and SB). All authors have extensive qualitative and co-design research experience with students and teachers in the school context. HG and SB are senior academics who are principle investigators, founders, and directors of LifeLab since 2018. LB and MM are PhD students and CP is a research assistant with LifeLab. This multidisciplinary team has expertise in education, pedagogy, behaviour change, psychology, and implementation science all of which informed the conceptualization, design, and conduct of this study.

CG is a postdoctoral researcher, who positions herself as a pragmatist, informed by elements of critical realism and transformative thinking. Her expertise lies within mixed methods and qualitative research in the field of school-based, adolescent physical activity promotion research. CG maintained reflexivity throughout the analytical and writing process by noting questions, thoughts, feelings, and interpretations of the data and debating these with co-authors. This included reflexive writing and collaborative reflection on personal, interpersonal, methodological, and contextual decisions and beliefs (Olmos-Vega *et al*. 2023). Co-authors (LB, CP, HG, and SB) played a mentorship role in the initial stages of reviewing the data whereby CG asked questions to understand the context of the data and the intervention when uncertainties arose. During the analysis co-authors (LB, HG, and SB) acted as ‘critical friends’ to discuss observations, assumptions, and encourage a nuanced perspective of the data (Smith and McGannon 2018).

### Analysis

Data sources were analysed independently and integrated at the point of write up by mapping to the Orsmond and Cohn framework (Fig. 2). Interviews and focus group data were analysed by CG using a combined inductive–deductive reflexive thematic analysis. This included an initial step of data driven coding followed by a second step of mapping the generated codes to an existing framework while embracing the knowledge and expertise of the analyst (Orsmond and Cohn 2015, Braun and Clarke 2019). Transcripts were pseudonymized before entered and analysed in NVivo (Version 15.3.2). CG read and re-read the transcripts for data familiarization. Iterative open coding was followed until all data in each transcript was provided with initial codes. Once semantic codes were generated from meaningful data extracts, the initial process of grouping similar codes into common thematic areas was conducted by CG to determine any patterns across the data (i.e. inductive). These groupings were then labelled to generate initial themes which were then presented to, and discussed with, members of the research team to gain a nuanced holistic perspective (LB, SB, and HG). The initial themes were then mapped to the five feasibility objectives of this study (i.e. deductive) by the CG using the guiding questions from Orsmond and Cohn (2015) appendix to determine the best fit of the data to each objective.

Survey data were entered to SPSS (Version 30), where cleaning, reverse scoring and coding of sum scores was completed. Descriptive statistics were run to describe the sample characteristics, percentages, means, and standard deviations. The internal consistency of the scales were assessed using Cronbach's alpha. All statistical analyses were completed by CG.

To integrate the various data sources, all evidence was examined and mapped to the corresponding feasibility objective by CG. The mapping of the data was guided by the specific guiding questions outlined in the appendix from Orsmond and Cohn (2015) to determine the match between the data source and the objective. All data were presented under each of the objectives to fully integrate all data sources within the results. The initial write up was completed by CG and checked by all co-authors to ensure fit and consistency between the data points and feasibility objectives.

## Results

Figure 2 outlines the various data sources and data that were collated and mapped to the Orsmond and Cohn (2015) objectives for feasibility studies. In this section, all data sources are combined and presented under the themes of the feasibility objectives. This includes quotes from the interviews and focus groups, percentages, means, and standard deviations from the survey data and key notes and percentages from the researcher records. The key findings and implications for research from each of the five objectives are summarized in Table 1.

**Table 1:** Summary of key findings and recommendations for future research.

Feasibility objective	Key findings	Implications for future research
Recruitment and sample characteristics	Recruitment at the school level was challenging but was acceptable at the individual level. Attrition rates were low. The sample had an even split across male and female and only one individual who reported ‘prefer not to say’. Students were all in their first and second year of school and were mainly aged 13 or 14 years. Overall, the LifeLab programme targeted the correct audience (i.e. junior cycle students from DEIS schools) and is suitable for any teacher to deliver. Some teachers felt more advanced learning content is needed for second year students.	Develop an effective school recruitment strategy by working with different school actors to establish what works, for whom, and in what context. Adapt to other age and population groups. Encourage any teacher comfortable with the interactive and discussion-based nature to deliver LifeLab, not only limited to those with a wellbeing teaching background.
Data collection procedures and outcome measures	All outcome measures had good internal consistency. However, survey administration required the skilled researchers’ to guide students through the scales and answer questions. Data completeness was good. Some teachers raised issues with the consent process and the difficulty of maintaining student attention during the surveys. Researchers visiting the school for data collection was an opportunity to provide implementation support.	Establish test–retest reliability of the primary and secondary outcome variables for adolescents from socioeconomically disadvantaged backgrounds. Survey administration requires at least two members of the research team to account for classroom needs and ensure data completeness. Work with school actors, University Research Ethics Committees and Data Protection Units to create inclusive and accessible consent processes. Utilize data collection as another opportunity for implementation support.
Acceptability and suitability of the intervention and study procedures	Teachers felt their students were able to engage with the content, it was appropriate, relevant and had sufficient detail. However, some optional additional content would be useful to have to hand. Strengths of LifeLab include its ease of delivery, positive impact on the students’ learning and development, student-centred nature, and Wellbeing curriculum alignment. Nonetheless some implementation challenges are faced during the classroom lessons, the visits to DCU and the photovoice project, but there are strategies to overcome these challenges.	Allow flexibility around intervention delivery for schools. Ensure the development of, and the intervention itself, are student-centred. Include co-designed vignettes more to encourage discussion of sensitive topics without making it too personal. Align with the school curriculum to increase buy-in. Utilize photovoice as a methodology for classroom-based assessments as it empowers students to learn about health in their natural environment. LifeLab is suitable for all types of learners due to the interactive and discussion-based nature.
Resources and ability to manage and implement the study and intervention	This study was manageable with two full time employees, many volunteers and project oversight for a total of four schools. Specific facilities and a protected space is required for the workshop visits. Schools funded their travel to attend workshop visits. There were some slight adaptations to the intervention material when delivered by individual teachers to fit their context and needs.	Carefully consider staffing and capacity required to run a full-scale definitive trial. Expect adaptations and allow flexibility to adapt intervention delivery rather than recording noncompliance or low fidelity. Include desirable and essential programme delivery criteria. Source emergency funding for schools to participate in the LifeLab workshops.
Preliminary evaluation of participant responses to intervention	Findings show evidence of promise that LifeLab could result in improved adolescents’ health literacy levels. Teachers felt that they would benefit from having extra information on each of the health topics (i.e. sleep, physical activity, food choices, substance misuse, mental health, socioeconomic factors) and activities in classroom lessons if the teacher has additional time to fill. Challenges faced during the photovoice project included group dynamics and difficulty interpreting the accompanying worksheet.	Include structured training for teachers. Potential to restrict access to programme materials until training is complete. Training should deliver information in different formats (i.e. videos, worked examples etc.). Ensure lessons can be delivered easily by teachers with little preparation time required. The option of additional content will give teachers autonomy to adapt to the context of needs in their classroom. Utilize the LifeLab visits as an opportunity to provide implementation support for teachers (otherwise it would be a remote programme). Need a fully powered study with a control group to determine effectiveness of LifeLab. Include optional extra information in the intervention content.

### Objective 1—Evaluation of recruitment capability and resulting characteristics

Of the 66 schools invited, four schools agreed to participate in this study including three single gender schools (two all girls, one all boys) and one mixed gender school. A total of 391 students, 15 teachers and one classroom assistant from the four participating schools were invited to participate in this study (Fig. 1). Only one mixed gender school agreed to participate in the student focus groups. A total of 13.8% and 16.8% were lost at postintervention and follow-up respectively (Supplementary File S4). Almost two-thirds of participants were 13 or 14 years old and there was an even split of male and female students (Table 2). At baseline, the majority of students had moderate HL levels (76.9%) and good wellbeing (63.4%) scores.

**Table 2:** Demographic characteristics and baseline scores of the participating students.

Characteristics	Category	% (*N* = 345)	Frequency^a^
Gender	Male	49.3	170
	Female	50.4	174
	Prefer not to say	0.3	1
Age	12	33	105
	13	36.8	117
	14	29.6	94
	15	0.6	2
Year group	1	52.3	180
	2	47.5	164
School type	Mixed	66.1	228
	Single gender—girls	22.3	77
	Single gender—boys	11.6	40
Follow-up duration	3 months	53	183
	6 months	47	162
Health literacy	Low	12	37
	Moderate	76.9	237
	High	11	34
Wellbeing	Poor	36.6	110
	Good	63.4	189

Scale	Total *N*	Mean	SD
Health literacy score	301	30.2	4.3
Wellbeing score	307	14.8	4.9
Physical health score	302	44.8	7.6
Psychological health score	305	44.8	9.2
Parental support and autonomy score	299	47.8	9.4
Peer support score	305	47.9	10.1
School learning and enjoyment score	303	44.3	8.3

^a^Where frequency does not equate to the total number of 345, the participants did not respond to this information

Students and teachers in LifeLab schools believed that LifeLab is targeted at the correct audience (i.e. Junior Cycle students). It is especially suitable for students ‘from areas of socioeconomic disadvantage… it ticks all of the boxes, all the important health issues that they would face on a daily basis.’ (Male teacher, School 4).

Teachers felt that LifeLab was most suitable ‘for first years’ (Male teacher, school 4). While teachers that delivered the programme to second and third year students explained the need for it to be more advanced as they have already learned about some of the topics in other subjects.

‘It was more tailored to probably a younger, slightly younger [audience]. I do think they [second years] could take more content in if I'm to be perfectly honest’ (Female teacher, School 4)

Teachers felt that LifeLab could be delivered by any teacher that is comfortable with the interactive and discussion-based nature of the lessons around sensitive health topics. However, they recognize that ‘there would be some teachers that would be very uncomfortable doing that’ (Male Teacher, School 2) and may require more support.

Teachers with a health and wellbeing background found it very easy to deliver but equally other teachers who did not were able to manage delivering the lessons without any issues.

‘I don’t have a health and wellbeing background in that I am not a PE [Physical Education] teacher or I am not a science teacher, I am not a home ec [economics] teacher so I wouldn’t have any of that sciency stuff …. and I could manage it’ (Female teacher, School 1)

### Objective 2—Evaluation and refinement of data collection procedures and outcome measures

The majority of the teachers raised some concerns about the research arm of the study in that the consent forms were ‘way too complicated, and it is why a couple of my parents wouldn’t’ engage with them (Female teacher, school 1). Another teacher suggested that it could be improved ‘if you could amalgamate the parent and the students’ consent forms (Male teacher, School 2). However, only 9% of students (*N* = 391) did not provide consent to participate in this study.

During piloting the surveys the researchers’ noted some student comprehension difficulties. For these reasons the researchers adopted a protocol whereby the HL and WHO-5 wellbeing index were read aloud by the researcher during the survey administration; however, students were able to complete Kidscreen-27 independently. The surveys were administered as per the published protocols but if a student asked for help during the survey the research team developed a script to respond to frequently asked questions (Supplementary File S5).

However, data completeness was good with <3% of participants excluded from analyses at baseline, <2% at postintervention, and less than one and a half percent at follow-up (Supplementary File S4). Those excluded accounted for cases where data within the scale was missing and a sum score could not be calculated. One teacher felt that the students ‘weren’t very attentive when they were filling’ the surveys out but that it could have been because ‘they were hyper, it was the end of the day, they were excited’ (Female teacher, School 2).

All outcome measures performed well in that the internal consistency of each of the seven scales and subscales were within acceptable range (i.e. Cronbach's alpha ≥.7) (Table 3). One teacher highlighted that the research visits were ‘a great way to set it [LifeLab] up’ for the students at the beginning (Male teacher, School 4).

**Table 3:** Performance of outcome measures and mean changes over time for total sample.

	Baseline				Postintervention				Follow-up			
	α	*N*	M	SD	α	*N*	M	SD	α	*N*	M	SD
Health literacy 10-item	0.77	301	30.18	4.30	0.8	306	31.68	4.28	.83	298	31.96	4.36
WHO 5-item wellbeing scale	0.78	307	14.78	4.94	0.80	307	14.51	4.85	.82	297	15.00	5.01
Kidscreen-27												
Physical wellbeing	0.74	302	44.81	7.64	0.79	302	44.60	8.20	.82	289	44.91	8.89
Psychological wellbeing	0.87	305	44.75	9.20	0.87	305	43.62	9.03	.90	291	44.45	9.77
Family and autonomy	0.85	299	47.84	9.37	0.87	301	48.00	10.32	.88	292	49.96	10.86
Peer support	0.83	305	47.94	10.09	0.85	305	47.45	10.11	.88	292	47.87	11.13
School environment	0.79	303	44.26	8.29	0.84	306	44.06	9.37	.85	292	43.28	9.01

### Objective 3—Evaluation of acceptability and suitability of the intervention and study procedures

The health topics taught through LifeLab were relevant for all students and the methods used were suitable ‘for all types of learners as well, if they're talking to their friends and they're discussing what kind of pictures, and then they're looking at the images, explaining them to the class’ (Female teacher, School 4). Furthermore, teachers emphasized the importance of wellbeing in the school setting and felt that LifeLab provided an opportunity for students to meaningfully learn about their health.

‘Well because wellbeing is such an important part of school, they [health topics] are definitely relevant and they [health topics] are definitely important. And I suppose some students won’t be doing home ec [economics] or won’t be doing science and while they will all be doing SPHE [Social, Personal, and Health Education] I think the information they get out of this is really important to reinforce it. So it is good that they do hear it from a number of areas because each time they hear it they will pick up something different.’ (Female Teacher, School 3).

Teachers determined the suitability of LifeLab for their students through observing the students’ understanding of the message imparted by the programme and the levels of engagement in the classroom. This was assessed through the teach-back method and the level of ‘feedback you are getting from them and then you will kind of know then if it is appropriate for them or not because then they either will be able to give the feedback or they won’t’ (Female teacher, School 1). In addition, teachers stated that students’ attendance on school trip days were usually better than normal school days and this was the same when they went to ‘DCU, nearly everyone was there’ (Male teacher, School 4).

Mostly, teachers found LifeLab easy or very easy to deliver (92.8%). The lesson plans were largely very good or excellent (85.7%), the scheme of work was very good or excellent (100%), and the powerpoints were very good or excellent (78.6%). Teachers really liked the LifeLab photovoice project (100% very good or excellent). They found it easy (35.7%) or very easy (57.1%) to deliver and all 14 said they would use this style of project again.

Teachers and students reported the positive impact of LifeLab on students’ learning and development. It helped students to develop transferable skills such as teamwork, communication, critical thinking, public speaking, and forming their own opinions.

The photovoice project helped students apply their skills and their learnings from LifeLab into practice. It helped them to notice the facilities that were available in their school that they had not noticed before such as the ‘wellbeing garden.. or the gym’ (focus group 6). Moreover, it encouraged natural health conversations as they ‘were walking around’ the school taking pictures they ‘would talk about, like, do you think like, it is to do with mental or physical health’ (focus group 8). Teachers really liked that the photovoice project naturally stimulated these health-related conversations.

‘It is taking those instructions, taking the photos and bringing it together for them to form their own opinion. I think that's great because, I mean, that's what we try to teach our students, is to kind of.. again, to be independent, but also for everyone then to reflect on it and teamwork’ (Male teacher, School 4).

Finally, the LifeLab environment was conducive to a positive, interactive learning experience outside of the classroom. Students felt comfortable to have those health-related conversations and ultimately solidified their learning.

‘The fact it is enjoyable, the fact that there's games, and I think the fact that the learning environment [in LifeLab] is so positive, I think that gets them [the students] to open up and maybe say things that they wouldn't usually feel comfortable saying to a teacher or someone about health issues and about their own experiences with, health topics… and just how enjoyable it is for them. It is a real positive experience for them and the takeaway is positive. So therefore, the messages I think are more likely to stick with them then afterwards.’ (Male teacher, School 2).

LifeLab encourages students to engage in self-directed learning and to complete a group project. It gives students autonomy and creativity over their learning about health and gives the teacher a facilitating role in that learning process.

‘It is [LifeLab] very student-led, I'd say, as opposed to me just talking about it all the time, it gives even though you still are talking quite a lot to them, you're posing the questions a lot. It is almost like discussion based’ (Male teacher, School 4).

There were many aspects that students and teachers liked about LifeLab such as, the photovoice project, the LifeLab characters, the groupwork, practical activities, getting out of the classroom, the resources and support provided by DCU, and the health topics.

In terms of groupwork during the photovoice project the students liked having peers around so they could discuss and ask each other questions such as ‘why do you think it [the object photographed] is there and stuff and like, what do you think is more important, like this one [photo] or that one, and what do you think of it?’ (focus group 8).

Both the students and the teachers valued the LifeLab visits to DCU; they felt that it helped the students see that ‘there was actually a point to it, as opposed to just going through the motions in school.’ (Male teacher, School 2). Teachers found that the resources ‘couldn’t be easier’ to understand and they did not feel burdened by the preparation for each lesson (Female teacher, School 3).

Students learn about the LifeLab health topics which were previously identified by young people in co-design work; sleep, physical activity, mental health or wellbeing, substance misuse, accessing health information, and diet which are all included in strand two of the Social, Personal, and Health Education curriculum. It also aligns with other subjects that cover health such as Physical Education, Science, and Home Economics so the topics are cross-curricula. However, LifeLab covers the topics ‘more in depth’ and from a different perspective than the other subjects.

‘[The content is] enough for them and like we do have more lessons in SPHE [Social, Personal, and Health Education] where we delve into more detail on exercise and things like that and it is done in other subjects as well. So it is not standalone, it is being supported by other subjects’ (Female teacher, School 3).

This was confirmed when the majority of teachers (92.9%) who responded to the survey felt that the LifeLab lessons were very or extremely relevant to the Social, Personal, and Health Education curriculum.

There were some challenges faced by teachers and students at different stages of the programme such as during the school-based lessons, LifeLab visits, or photovoice project. However, some strategies to overcome these implementation challenges were suggested such as implementing teacher training.

For the most part, teachers felt that their students were engaged during the classroom lessons, although you will always have ‘one or two students who don’t engage much across the board’ (Male teacher, School 2). One teacher highlighted that ‘it is not that they [the students] won’t talk because they are being rude, far from it, they are afraid of being wrong’ (Female teacher, School 1).

Some students reported that they did not like the lessons when they ‘just have to sit in a class and just look at a whiteboard for an hour’ (focus group 3) or when they ‘just needed to write stuff down about the characters [vignettes]’ (focus group 4).

In the survey teachers rated both visits to LifeLab very good or excellent (100%) and they would all recommend LifeLab to other teachers. However, during the teacher interviews they mentioned that there were some challenges faced during the interactive LifeLab workshop visits.

No challenges were reported by the teachers during the interactive workshops but two teachers from one school reported issues associated with travelling to the university. This school had to travel with large groups of students on the public bus and they would benefit from ‘funding for a bus because we really struggle to get here’ (Female teacher, School 1). This caused some additional stress for the teachers in this school as they ‘were a little bit more going like oh my god you’re [going] to get over to DCU, worry about the trips and the [consent] slips and the whole lot’ (Female teacher, School 1).

For the students the only challenges faced during the workshop was when they did not get to try each different aspect at a station in the lab for example, ‘when I didn't get to do the sleeping thing’ or ‘when I didn't get to do the boxing thing’ (focus group 7).

Some of the challenges faced during the photovoice project were groupwork dynamics, the students felt ‘there wasn't a lot to take pictures of’ (focus group 10), some of the questions on the worksheet ‘confused them [the students] a lot’ (Female teacher, School 1), the use of technology, and the presentation of the projects.

‘When I got them to stand up and try and explain why they chose the picture, or, you know, kind of go through the five questions with them, they were a little bit kind of, I don’t know, maybe lost in that situation. I suppose they are in their first year.’ (Male teacher, School 4).

There were some suggestions to improve programme implementation by embedding strategies to overcome the challenges. Teachers felt that adding some teacher training before they deliver LifeLab would be helpful for them and having different formats to get the information across not just ‘paperwork’ including ‘videos of how it looks in the classroom’ would be helpful (Female teacher, School 1).

‘for the teachers who are doing Lifelab for the first time to do an in-service [training] before they do it… even an online webinar’ (Female teacher, School 1).

In relation to maximizing student engagement, students suggested including more practical and engaging lessons for example, ‘more videos rather than reading a PowerPoint’ (focus group 8) or a ‘kahoot’ quiz (focus group 4). Teachers felt that having ‘more fun, applied or active’ lessons can naturally facilitate health discussions ‘without actually having to bring it up’ (Male teacher, School 2). Teachers felt they could encourage this by ‘maybe kind of looking at the characters [vignettes] a little more because they did really like looking at the characters’ (Female teacher, School 4) or by the teacher really encouraging the class to engage in discussions.

Fifty-seven percent of teachers that responded to the survey (*N* = 14) evaluating LifeLab indicated that they were able to complete all of the seven school-based lessons. The lessons not completed included the first introductory lesson (*n* = 1), lesson three meeting the LifeLab characters (*n* = 1), the trial project lesson four (*n* = 2), and the final closure lesson (*n* = 2).

As the programme was delivered by teachers in different schools, there were nuanced styles of delivery. In some instances, the programme fidelity was lower for example, it was not delivered during the intended Social, Personal, and Health Education class time rather during a different subject that the same teacher had (i.e. Physical Education or Science). Another example of this:

‘So I intentionally skipped one topic in the slides and it was about the substance abuse video that was on show because I had a look at it myself beforehand… So it was just one choice I made where I said this actually isn’t relevant and I’m under time pressure because slips and permission and all the fiddly paperwork so that was just one thing.’ (Female teacher, School 1).

Finally, one key piece of feedback from the students was that during the photovoice project the teacher deviated from the guidelines whereby the teacher ‘picked the pictures [for the students to discuss and write about] if they were appropriate’ (focus group 3) and they were ‘given out randomly in a group so you wouldn’t have got your [own] photo’ (focus group 4). This affected the way students answered the worksheet questions as ‘some of the photos were never related to any of the questions, so it was kind of hard to give your opinion on it’ (focus group 4).

Other slight deviations included different strategies used to pick the student groups, the location chosen to take pictures for the project, and teachers getting students to write instead of discuss points.

The photovoice project worked whereby students went around the school or in some cases their community to take pictures of ‘Stuff that relates to health, mental health, physical health’ (focus group 1). Students used a phone or an iPad to take pictures of a variety of things such as the ‘PE gym’, ‘a photowall full of quotes’, ‘the wellbeing garden’, ‘vapes…that were on the floor’, ‘empty cans’, ‘fast gas bottles’, etc. Some class groups worked in small groups while others did the activity individually. Students engaged in ‘teamwork’ (focus group 2) and communication to decide which photos their group would use. When they returned back to the classroom in their groups they ‘talked about why we chose these photos’, ‘what the photos were, what was in it, basically the subject matter’ (focus group 2). Finally, the students presented their projects in their groups back to their class.

### Objective 4—Evaluation of resources and ability to manage and implement the study and intervention

The DCU LifeLab team consisted of researchers at different career levels (i.e. principal investigators, postdoctoral researchers, PhD students, and research assistants). Throughout the duration of this study there were two full time researchers (LB and CP) who managed consent procedures, data collection, inputting, analysis, and running the LifeLab visits. In addition, undergraduate volunteers from the Physical Education, Sport Science and Health, and Health and Society programmes facilitated the LifeLab workshops. Each workshop included a minimum of five and maximum of ten facilitators and one leader. Undergraduate facilitators are integral to the implementation of the LifeLab workshops in DCU. Semester one proved difficult to recruit undergraduate facilitators due to demanding timetables; however, Semester two was slightly easier. Thus, the reduced capacity in semester one may have hindered the fidelity of the intervention as the workshops had to operate at a reduced capacity. Study management and oversight was managed by the principle investigators (HG and SB).

The space and equipment available to deliver the LifeLab programme was integral especially during the workshops at DCU. The university had the designated space to conduct the LifeLab visits which could host a maximum of 30 school students for one workshop which removed the barrier of cost. There was a lot of equipment required for the LifeLab workshops including powerpoints, games-based equipment, and iPads which were largely budget friendly however, would not have been possible without the support of research funding. The remainder of the intervention was conducted during normal class time in each school, delivered by the class teacher. Classroom resources included powerpoints, lesson plans, and classroom handouts which were provided to each teacher via google drive and the teacher printed resources where necessary for each lesson.

The research funding was available to cover the PhD student stipend, the research assistant salary, travel to and from schools for data collection, purchasing of equipment for LifeLab, and printing of surveys and consent forms.

### Objective 5—Preliminary evaluation of participant responses to intervention

While the feedback about the LifeLab programme was largely positive, there were some suggestions from teachers and students to improve the programme content further by maximizing timing of the lessons, adding more in-depth information to the lessons, making the lessons more interactive and engaging, and improving the structure and design of the lessons.

Additionally, the main feedback on the resources for the programme included ‘more time at the project’ (Female teacher, School 1) and more materials to fill any extra time during classes.

Teachers felt that there was room for improvement in the photovoice project which could be achieved through more clear teacher instructions, project adaptations, and participation formation. Teachers wanted clear, structured but flexible guidance to facilitate the delivery of the project. Including options to progress the project further would be beneficial for example, ‘bring them [students] out a little bit further in the community..like going to the cafe and looking at the menu and taking a few photos and really analysing… the menu and stuff’ (Female teacher, School 1). Further suggestions to improve the students’ experience included, allowing flexibility for students to either work in groups or alone and deciding whether they want to present the project or not were important factors to consider.

‘I don't know if you'd necessarily ask everyone [to present], but I think it could be like that two groups would be selected and you could pick the willing contenders… But I think definitely having something tangible that they get to deliver. I think they'll be more inclined to… engage with it a little bit more’ (Male teacher, school 2).

The LifeLab health topics covered in the programme were all deemed relevant and important however, feedback from teachers and students highlighted the need to add some more information on these topics, particularly around sleep, substance misuse, and accessing health information. Almost all teachers (92.9%) believed that the LifeLab health topics covered were very or extremely relevant for their students, and 85.7% of teachers found that the lessons were very or extremely engaging for students. Over half of teachers (64.3%) reported that the health topics were covered in enough detail in the lessons. Some teachers were looking for more information on topics such as ‘substance misuse’, ‘cyber bullying’, ‘friendships’, ‘smart devices’, ‘effects on social media on mental health or wellbeing’, or ‘stress’.

The examination of the preliminary impact of the LifeLab programme on adolescents’ HL, wellbeing, and health-related quality of life showed some evidence of promise. Baseline descriptive statistics for the overall sample are reported in Table 2 above. Overall, means and standard deviations showed improvements in HL, physical health and parental support and autonomy at postintervention and at follow-up (Table 3). There were overall decreases in adolescents’ self-reported psychological wellbeing, peer support, and school and learning from baseline to postintervention and follow-up. Adolescent wellbeing scores decreased from baseline to postintervention but increased beyond baseline levels at follow-up. Table 4 provides a breakdown of the average responses by gender and year group. In general, females reported better HL levels at baseline and follow-up whereas, males reported better wellbeing and health-related quality of life across all timepoints. Students in their second year of school generally reported higher HL, wellbeing, and health-related quality of life than those in first year.

**Table 4:** Comparing means and standard deviations by gender^a^ and year group across timepoints.

		Males			Females			Year 1			Year 2		
Outcome measure	Timepoint	*N*	M	SD	*N*	M	SD	*N*	M	SD	*N*	M	SD
Health literacy	Baseline	158	30.2	4.28	149	30.26	4.38	162	30.08	4.49	145	30.37	4.15
	Postintervention	157	31.69	4.08	152	31.62	4.47	160	31.15	4.45	148	32.19	4.03
	Follow-up	155	31.72	4.36	144	32.16	4.38	157	31.59	4.4	142	32.25	4.28
Wellbeing	Baseline	161	15.96	4.62	152	13.51	4.94	167	13.98	4.93	146	15.68	4.78
	Postintervention	157	15.49	4.71	153	13.43	4.81	160	14	5.0	149	14.97	4.68
	Follow-up	154	16.08	4.47	144	13.88	5.3	156	14.81	5.27	142	15.18	4.7
Physical wellbeing	Baseline	158	46.49	6.82	150	42.89	7.98	164	45.19	7.86	144	44.27	7.31
	Postintervention	154	46.92	7.38	151	42.22	8.3	157	43.96	8.75	147	45.21	7.51
	Follow-up	150	46.68	8.24	140	43.01	9.19	157	43.96	8.75	135	45.1	8.66
Psychological wellbeing	Baseline	162	47.1	8.78	149	42.12	8.92	165	44.93	9.02	146	44.43	9.39
	Postintervention	155	45.54	9.07	153	41.57	8.51	159	43.3	9.05	148	43.75	8.92
	Follow-up	150	46.59	9.25	142	42.2	9.77	156	44.89	9.83	136	43.66	9.33
Parents and free time	Baseline	155	49.55	8.68	150	46.1	9.57	164	46.35	8.96	141	49.33	9.17
	Postintervention	153	49.7	9.08	151	46.19	11.13	155	47.09	10.39	148	48.67	9.95
	Follow-up	137	51.27	10.33	127	48.59	11.25	146	49.35	10.79	118	50.44	10.77
Friends	Baseline	159	49.0	9.26	152	46.7	10.7	165	48.52	9.96	146	47.21	10.10
	Postintervention	155	47.62	8.98	153	47.24	11.13	159	47.77	10.5	148	46.94	9.56
	Follow-up	150	48.49	10.28	142	47.22	11.97	157	47.72	10.61	135	47.86	11.65
School and learning	Baseline	160	44.4	7.66	149	43.76	8.97	163	45.09	8.03	139	42.96	8.52
	Postintervention	156	45.01	8.76	143	42.95	9.84	160	44.14	9.21	148	43.77	9.53
	Follow-up	151	44.04	8.36	142	42.49	9.58	157	43.89	8.9	136	42.61	9.07

^a^One participant preferred not to state their gender and thus is not included in this table.

## Discussion

This LifeLab Dublin study was evaluated using the five key objectives for feasibility studies (Orsmond and Cohn 2015). Across these objectives, findings indicated feasibility outcomes across recruitment capabilities and sample characteristics; data collection procedures and outcome measures; acceptability and suitability of the intervention and study; resources and ability to manage and implement the study and the preliminary evaluation of participant responses. In summary, appropriate participants were recruited and the retention was high. The data collection procedures and outcome measures were suitable for the participants and for measuring the intended outcome. Participants had a positive experience with the LifeLab programme, its content, and materials, but have suggested some strategies to overcome potential barriers to implementation. The research team had sufficient capacity to manage the study demands and the programme fidelity was acceptable. Finally, these findings highlight evidence of promise for meaningful improvement in adolescents’ HL levels.

While recruitment at the school level was low (6%), this could be explained by the method of contacting schools via their generic email as opposed to reaching out to individual teachers. Additionally, contact was initiated by a PhD student which may have introduced a power dynamic. Furthermore, schools are busy, complex, and adaptive subsystems and the schools in this study have an added socioeconomic disadvantaged background which may present additional challenges when it comes to engaging in external opportunities (Daly-Smith *et al*. 2020).

In this current study, the attrition rates were consistent with similar studies, which have reported an average attrition rate of 16.2% postintervention (range 0%–54.1%) and 13% at follow-up (Hynynen *et al*. 2016), compared to 13.8% and 16.2%, respectively in this study. Nevertheless, attrition rates should be interpreted with caution due to varying participant and intervention characteristics, school contexts, study designs, and follow-up duration (Henneberger *et al*. 2023). This study demonstrated a high consent rate (91%) in comparison to that of similar studies in the literature, despite consent being expressed as a challenge for the schools in this study. For example, the GoActive and physically active lessons feasibility studies in the UK reported 78% and 82.5% consent rates, respectively (Corder *et al*. 2016, Gammon *et al*. 2019). Another, larger, randomized controlled feasibility study in the UK reported a consent rate of 34% among 9 to 11 year olds (Jago *et al*. 2014).

The data collection procedures followed in this study achieved good response rates and high levels of data completeness. This is comparable to survey data completeness in other school-based pilot or feasibility studies (Bonell *et al*. 2015, Hankonen *et al*. 2017). All teachers participated in the interviews and only one teacher did not complete the online survey. Student survey participation rates were good but only one of the three schools participated in the focus groups. These interviews and focus groups took place during the scheduled LifeLab visits and thus, did not require teachers or students to give-up any extra time. The student survey in this study was estimated to take ∼20 min which aligns with the recommended time for this target population group of ≤10–20 min (Omrani *et al*. 2019). Furthermore, surveys were administered by members of the research team during class time and were completed via pen and paper, which has been previously linked to less missing data than online or ‘self-administered’ surveys for adolescents (Denniston *et al*. 2010). All of which may have contributed to the good levels of data completeness (Supplementary File S4).

The outcome measures performed well for their intended purpose, which was expected as all scales were developed for, and previously validated in, this population age group (Ravens-Sieberer *et al*. 2007, Paakkari *et al*. 2016, Sischka *et al*. 2026). Furthermore, the research team highlighted some issues faced with particular items when administering the survey to students and provided suggestions to rephrase the items when asked (Supplementary File S5). However, this study represents adolescents attending socioeconomically disadvantaged schools in Dublin thus, the reliability and validity of these measures for this population should be established before proceeding to a definitive trial.

A systematic review examining health-related interventions in disadvantaged schools highlighted three key effective intervention strategies including ‘hands on’ practical activities, peer support, and holistic approaches (Smith *et al*. 2021). LifeLab upholds these strategies through the use of practical in-person visits to the university, undergraduate student facilitators, groupwork, and the photovoice project in the school, which has ultimately led to the satisfactory participant responses in this study. Students and teachers reported that the engaging, interactive, and student-centred nature were some of the many aspects they enjoyed about the programme. However, some areas for improvement noted were to include more practical activities during the school-based lessons to keep students engaged, difficulties travelling to LifeLab at DCU, and group dynamics during the photovoice project. Logistical challenges such as funding for travel and barriers to implementation including a lack of staff or management buy-in, competing priorities, and limited capacity or knowledge of programme implementers are commonly reported in school-based health interventions (McHale *et al*. 2022, Schäfer *et al*. 2025).

This LifeLab Dublin study was manageable for the research team due to the mix of expertise and hands-on practical support from undergraduate student volunteers, PhD students, research staff, and principle investigators. Furthermore, the school-based element of the programme was delivered by the teachers at each site, which coincides with ‘real-world’ circumstances. This approach is encouraged to enhance the long-term sustainability of interventions and reduces overall cost for later intervention scale-up (Lane *et al*. 2021). Nonetheless, ‘real-world’ implementation must consider the individual school context and accept that adaptations to the intervention are necessary to suit the school context and ensure successful implementation (Jago *et al*. 2023). One of the key success factors for implementation of LifeLab was the importance of flexibility when delivering LifeLab for teachers. Thus, future recommendations include providing teacher training and ongoing implementation support. Another key factor that needs to be considered for the future sustainability of LifeLab is the feasibility of schools travelling to the LifeLab facility at DCU. Finally, the potential for future national scale-up needs further consideration and exploration as the current DCU-based model may not be accessible for disadvantaged schools across Ireland.

Finally, this study demonstrates evidence of promise that LifeLab may improve adolescents’ HL levels with increases observed from baseline to postintervention and from postintervention to follow-up. This finding, as well as the good internal consistency of the scale, indicates that HL is a suitable primary outcome measure to evaluate the impact of the LifeLab programme in a definitive trial. There are few health-related school-based interventions that target or use HL as an outcome measure which makes comparison to the literature challenging (Smith *et al*. 2021). The LifeLab Southampton pilot cluster randomized control trial which also targeted second level school students’ HL, demonstrated improvements in adolescents’ knowledge of health and disease concepts, and the influence of behaviours on their long-term health and that of their children (Woods-Townsend *et al*. 2018). However, the aforementioned study used a different instrument to that in the current study. A more comprehensive evaluation on the impact of LifeLab on adolescents’ HL and wellbeing is ongoing and will be reported elsewhere.

### Strengths and limitations

The use of mixed methods and various data sources strengthens the quality of our findings due to the diverse range of feedback gathered and rigorous evaluation of the programme. In addition, CG provided a different perspective to the programme being a new member to the team in comparison to the remaining authors who were involved in the conceptualization of the study. This provided a more nuanced view of the intervention feedback, its strengths, and areas for improvement.

The findings should be interpreted in light of some limitations. Although all eligible schools across Dublin were invited, additional recruitment strategies were implemented by the research team (i.e. utilizing existing connections in schools) thus, it is unclear whether these schools would have participated had that connection not been present. Feedback from the student focus groups were only collected from one school which may not accurately reflect all students’ experiences within the programme.

## Conclusion

LifeLab Dublin is a feasible and acceptable study for students and teachers in socioeconomic disadvantaged schools across Dublin. Strengths of the LifeLab programme include its positive impact on students’ learning and development, the fact that it is student-centred, its alignment with the wellbeing curriculum, and the overall positive experience of LifeLab for students and teachers. There are some areas for improvement in LifeLab such as the logistics of running the photovoice project and the in-person visits to LifeLab, some optional extra information in the classroom-based lessons, and finally to integrate teacher training into LifeLab. Key feedback indicates that LifeLab can proceed to a definitive trial with some minor changes to the intervention and study procedures. A series of recommendations are provided for the future development, implementation, and evaluation of school-based HL interventions specifically with regards to intervention content and design, study design, recruitment and participation, and implementation support.

## Supplementary Material

daag127_Supplementary_Data

## Data Availability

The data underlying this article will be shared on reasonable request to the corresponding author.

## References

[daag127-B1] Bay JL, Vickers MH, Mora HA et al Adolescents as agents of healthful change through scientific literacy development: a school-university partnership program in New Zealand. Int J STEM Educ 2017;4:15. 10.1186/s40594-017-0077-0 30631671 PMC6310384

[daag127-B2] Bonell C, Fletcher A, Fitzgerald-Yau N et al Initiating change locally in bullying and aggression through the school environment (INCLUSIVE): a pilot randomised controlled trial. Health Technol Assess 2015;19:1–viii. 10.3310/hta19530 PMC4780984 26182956

[daag127-B3] Braun V, Clarke V. Reflecting on reflexive thematic analysis. Qual Res Sport Exerc Health 2019;11:589–97. 10.1080/2159676X.2019.1628806

[daag127-B4] Burke L., Gavigan N., Belton S. et al Health in our school: a photovoice project. Health Education. 2026;126:517–533. 10.1108/HE-09-2025-0179

[daag127-B5] Corder K, Brown HE, Schiff A et al Feasibility study and pilot cluster-randomised controlled trial of the GoActive intervention aiming to promote physical activity among adolescents: outcomes and lessons learnt. BMJ Open 2016;6:e012335. 10.1136/bmjopen-2016-012335 PMC5129050 27836873

[daag127-B6] Daly-Smith A, Quarmby T, Archbold VSJ et al Using a multi-stakeholder experience-based design process to co-develop the Creating Active Schools Framework. Int J Behav Nutr Phys Act 2020;17:13. 10.1186/s12966-020-0917-z 32028968 PMC7006100

[daag127-B7] Denniston MM, Brener ND, Kann L et al Comparison of paper-and-pencil versus Web administration of the Youth Risk Behavior Survey (YRBS): participation, data quality, and perceived privacy and anonymity. Comput Hum Behav 2010;26:1054–60. 10.1016/j.chb.2010.03.006

[daag127-B8] Department of Education and Skills . Post-primary Schools Enrolment Figures 2024/2025. Dublin: Ireland. Government of Ireland, 2025. https://www.gov.ie/en/department-of-education/collections/post-primary-schools-enrolment-figures/

[daag127-B9] Eldridge SM, Chan CL, Campbell MJ et al CONSORT 2010 statement: extension to randomised pilot and feasibility trials. Br Med J 2016;355:i5239. 10.1136/bmj.i5239 27777223 PMC5076380

[daag127-B10] Gammon C, Morton K, Atkin A et al Introducing physically active lessons in UK secondary schools: feasibility study and pilot cluster-randomised controlled trial. BMJ Open 2019;9:e025080. 10.1136/bmjopen-2018-025080 PMC6527971 31064805

[daag127-B11] Goss HR, McDermott C, Hickey L et al Understanding disadvantaged adolescents' perception of health literacy through a systematic development of peer vignettes. BMC Public Health 2021;21:593. 10.1186/s12889-021-10634-x 33765994 PMC7992854

[daag127-B12] Goss HR, Smith C, Hickey L et al Using co-design to develop a health literacy intervention with socially disadvantaged adolescents. Int J Environ Res Public Health 2022;19:4965. 10.3390/ijerph19094965 35564357 PMC9103747

[daag127-B13] Hankonen N, Heino MTJ, Hynynen ST et al Randomised controlled feasibility study of a school-based multi-level intervention to increase physical activity and decrease sedentary behaviour among vocational school students. Int J Behav Nutr Phys Act 2017;14:37. 10.1186/s12966-017-0484-0 28327174 PMC5361824

[daag127-B14] Henneberger AK, Rose BA, Feng Y et al Estimating student attrition in school-based prevention studies: guidance from state longitudinal data in Maryland. Prev Sci 2023;24:1035–45. 10.1007/s11121-023-01533-1 37195597 PMC10409654

[daag127-B15] Hynynen S-T, van Stralen MM, Sniehotta FF et al A systematic review of school-based interventions targeting physical activity and sedentary behaviour among older adolescents. Int Rev Sport Exerc Psychol 2016;9:22–44. 10.1080/1750984X.2015.1081706 26807143 PMC4706019

[daag127-B16] Jago R, Salway R, House D et al Rethinking children’s physical activity interventions at school: a new context-specific approach. Front Public Health 2023;11:1149883. 10.3389/fpubh.2023.1149883 37124783 PMC10133698

[daag127-B17] Jago R, Sebire SJ, Davies B et al Randomised feasibility trial of a teaching assistant led extracurricular physical activity intervention for 9 to 11 year olds: action 3:30. Int J Behav Nutr Phys Act 2014;11:114. 10.1186/s12966-014-0114-z 25209323 PMC4172904

[daag127-B18] Lane C, McCrabb S, Nathan N et al How effective are physical activity interventions when they are scaled-up: a systematic review. Int J Behav Nutr Phys Act 2021;18:16. 10.1186/s12966-021-01080-4 33482837 PMC7821550

[daag127-B19] Leger SL, Nutbeam D. A model for mapping linkages between health and education agencies to improve school health. J Sch Health 2000;70:45–50. 10.1111/j.1746-1561.2000.tb07239.x 10715824

[daag127-B20] McHale F, Ng K, Scanlon D et al Implementation evaluation of an Irish secondary-level whole school programme: a qualitative inquiry. Health Promot Int 2022;37:daac131. 10.1093/heapro/daac131 36287522

[daag127-B21] National Council for Curriculum and Assessment . Junior Cycle Wellbeing Guidelines. Dublin: IrelandDepartment of Education, 2021. https://ncca.ie/media/5062/updated-guidelines-2021_en.pdf

[daag127-B22] National Council for Curriculum and Assessment & Department of Education . Short Course: Social, Personal & Health Education (SPHE) Specification for Junior Cycle. Dublin: Ireland. Government of Ireland, 2023. https://www.curriculumonline.ie/getmedia/2780c6c6-993c-46fb-8ba6-b647188337c9/JC-SPHE-Short-Course-2023-EV-FINAL.pdf

[daag127-B23] Nutbeam D . Health literacy as a public health goal: a challenge for contemporary health education and communication strategies into the 21st century. Health Promot Int 2000;15:259–67. 10.1093/heapro/15.3.259

[daag127-B24] Nutbeam D, Muscat D. Health Literacy in a Nutshell. Sydney: Australia. McGraw-Hill Education Pty Limited, 2023.

[daag127-B25] Olmos-Vega FM, Stalmeijer RE, Varpio L et al A practical guide to reflexivity in qualitative research: AMEE Guide No. 149. Med Teach 2023;45:241–51. 10.1080/0142159X.2022.2057287 35389310

[daag127-B26] Omrani A, Wakefield-Scurr J, Smith J et al Survey development for adolescents aged 11–16 years: a developmental science based guide. Adolesc Res Rev 2019;4:329–40. 10.1007/s40894-018-0089-0

[daag127-B27] Orsmond GI, Cohn ES. The distinctive features of a feasibility study: objectives and guiding questions. Occup Ther J Res 2015;35:169–77. 10.1177/1539449215578649 26594739

[daag127-B28] Paakkari L, Okan O. Health literacy—talking the language of (school) education. HLRP: Health Lit Res Pract 2019;3:e161–4. 10.3928/24748307-20190502-01 31410386 PMC6685510

[daag127-B29] Paakkari O, Torppa M, Kannas L et al Subjective health literacy: development of a brief instrument for school-aged children. Scand J Public Health 2016;44:751–7. 10.1177/1403494816669639 27655781

[daag127-B30] Patton GC, Sawyer SM, Santelli JS et al Our future: a Lancet commission on adolescent health and wellbeing. Lancet 2016;387:2423–78. 10.1016/s0140-6736(16)00579-1 27174304 PMC5832967

[daag127-B31] Pearson M, Chilton R, Wyatt K et al Implementing health promotion programmes in schools: a realist systematic review of research and experience in the United Kingdom. Implement Sci 2015;10:149. 10.1186/s13012-015-0338-6 26510493 PMC4625879

[daag127-B32] Ravens-Sieberer U, Auquier P, Erhart M et al The KIDSCREEN-27 quality of life measure for children and adolescents: psychometric results from a cross-cultural survey in 13 European countries. Qual Life Res 2007;16:1347–56. 10.1007/s11136-007-9240-2 17668292

[daag127-B33] Sawyer S, Afifi AR, Bearinger HL et al Adolescence: a foundation for future health. Lancet (London, England) 2012;379:1630–1640. 10.1016/S0140-6736(12)60072-5 22538178

[daag127-B34] Sawyer SM, Azzopardi PS, Wickremarathne D et al The age of adolescence. Lancet Child Adolesc Health 2018;2:223–8. 10.1016/S2352-4642(18)30022-1 30169257

[daag127-B35] Schäfer SK, Streit S, Schäfer CG et al Barriers and facilitators for the implementation of preventative mental health interventions among secondary schools in high-income countries: a systematic review. Eur Child Adolesc Psychiatry 2025;34:3713–31. 10.1007/s00787-025-02796-5 40586958 PMC12743069

[daag127-B36] Sischka PE, Martin G, Abdeta C et al Cross-national validation of the WHO-5 well-being index within adolescent populations: findings from 43 countries. Assessment 2026;33:3–26. 10.1177/10731911241309452 39884717

[daag127-B37] Skivington K, Matthews L, Simpson SA et al A new framework for developing and evaluating complex interventions: update of Medical Research Council guidance. BMJ. 2021;374:n2061. 10.1136/bmj.n2061 34593508 PMC8482308

[daag127-B38] Smith B, McGannon KR. Developing rigor in qualitative research: problems and opportunities within sport and exercise psychology. Int Rev Sport Exerc Psychol 2018;11:101–21. 10.1080/1750984X.2017.1317357

[daag127-B39] Smith C, Belton S, Issartel J et al LifeLab Dublin; a process evaluation of a pilot health literacy intervention for socioeconomically disadvantaged adolescents. Curriculum Studies in Health and Physical Education. 2024;17:149–168. 10.1080/25742981.2024.2399010

[daag127-B40] Smith C, Goss HR, Issartel J et al Health literacy in schools? A systematic review of health-related interventions aimed at disadvantaged adolescents. Children 2021;8:176. 10.3390/children8030176 33668861 PMC7996245

[daag127-B41] Smith C, Goss HR, Issartel J et al LifeLab: co-design of an interactive health literacy intervention for socioeconomically disadvantaged adolescents’. Children 2022;9:1230. 10.3390/children9081230 36010120 PMC9406774

[daag127-B42] Story M, Nanney MS, Schwartz MB. Schools and obesity prevention: creating school environments and policies to promote healthy eating and physical activity. Milbank Q 2009;87:71–100. 10.1111/j.1468-0009.2009.00548.x 19298416 PMC2879179

[daag127-B43] Tang K, Nutbeam D, Aldinger C et al Schools for health, education and development: a call for action. Health Promot Int 2009;24:68–77. 10.1093/heapro/dan037 19039034

[daag127-B44] WHO, (World Health Organisation) . Shanghai declaration on promoting health in the 2030 Agenda for Sustainable Development. Health Promot Int 2017;32:7–8. 10.1093/heapro/daw103 28180270

[daag127-B45] Woods-Townsend K, Leat H, Bay J et al LifeLab Southampton: a programme to engage adolescents with DOHaD concepts as a tool for increasing health literacy in teenagers –a pilot cluster-randomized control trial. J Dev Orig Health Dis 2018;9:475–80. 10.1017/S2040174418000429 30101731 PMC6159871

[daag127-B46] World Health Organization . Global Accelerated Action for the Health of Adolescents (AA-HA!): Guidance to Support Country Implementation. Geneva: World Health Organization. WHO, 2017.

