# Supplementary material for: Feasibility of LifeLab Dublin: a co-designed health literacy intervention for socioeconomic disadvantaged schools
Source: Health Promot Int. 2026 Aug 18;41(4):daag127. doi: 10.1093/heapro/daag127 (PMC13483984; doi:10.1093/heapro/daag127)
Supplement: Supplementary file 1 [file daag127_supplementary_data.zip › Supplementaryfile1.docx]

**Supplementary file 1- CONSORT - pilot and feasibility trials checklist**

| **Section/ Topic** | **Item No** | **Checklist item** | **Reported on page No** |
| --- | --- | --- | --- |
| **Title and abstract** | | | |
|  | **1a** | Identification as a pilot or feasibility randomised trial in the title | **1** |
|  | **1b** | Structured summary of pilot trial design, methods, results, and conclusions (for specific guidance see CONSORT abstract extension for pilot trials) | **1** |
| **Introduction** | | | |
| **Background and objectives** | **2a** | Scientific background and explanation of rationale for future definitive trial, and reasons for randomised pilot trial | **1-2** |
|  | **2b** | Specific objectives or research questions for pilot trial | **2** |
| **Methods** | | | |
| **Trial design** | **3a** | Description of pilot trial design (such as parallel, factorial) including allocation ratio | **2** |
|  | **3b** | Important changes to methods after pilot trial commencement (such as eligibility criteria), with reasons | **N/A** |
| **Participants** | **4a** | Eligibility criteria for participants | **2** |
|  | **4b** | Settings and locations where the data were collected | **2-3** |
|  | **4c** | How participants were identified and consented | **2-3** |
| **Interventions** | **5** | The interventions for each group with sufficient details to allow replication, including how and when they were actually administered | **2-3** |
| **Outcomes** | **6a** | Completely defined prespecified assessments or measurements to address each pilot trial objective specified in 2b, including how and when they were assessed | **4** |
|  | **6b** | Any changes to pilot trial assessments or measurements after the pilot trial commenced, with reasons | **N/A** |
|  | **6c** | If applicable, prespecified criteria used to judge whether, or how, to proceed with future definitive trial | **N/A** |
| **Sample size** | **7a** | Rationale for numbers in the pilot trial | **2-3** |
|  | **7b** | When applicable, explanation of any interim analyses and stopping guidelines | **N/A** |
| **Randomisation:** |  |  |  |
| **Sequence**  **generation** | **8a** | Method used to generate the random allocation sequence | **N/A** |
|  | **8b** | Type of randomisation(s); details of any restriction (such as blocking and block size) | **N/A** |
| **Allocation**  **concealment**  **mechanism** | **9** | Mechanism used to implement the random allocation sequence (such as sequentially numbered containers), describing any steps taken to conceal the sequence until interventions were assigned | **N/A** |
| **Implementation** | **10** | Who generated the random allocation sequence, who enrolled participants, and who assigned participants to interventions | **N/A** |
| **Blinding** | **11a** | If done, who was blinded after assignment to interventions (for example, participants, care providers, those assessing outcomes) and how | **N/A** |
|  | **11b** | If relevant, description of the similarity of interventions | **N/A** |
| **Statistical methods** | **12** | Methods used to address each pilot trial objective whether qualitative or quantitative | **4-5** |
| **Results** | | | |
| **Participant flow** | **13a** | For each group, the numbers of participants who were approached and/or assessed for eligibility, randomly assigned, received intended treatment, and were assessed for each objective | **3** |
|  | **13b** | For each group, losses and exclusions after randomisation, together with reasons | **3, supplementary file 4** |
| **Recruitment** | **14a** | Dates defining the periods of recruitment and follow-up | **3** |
|  | **14b** | Why the pilot trial ended or was stopped | **N/A** |
| **Baseline data** | **15** | A table showing baseline demographic and clinical characteristics for each group | **7** |
| **Numbers analysed** | **16** | For each objective, number of participants (denominator) included in each analysis. If relevant, these numbers should be by randomised group | **3, 7, 8, 11, supplementary file 4** |
| **Outcomes and estimation** | **17** | For each objective, results including expressions of uncertainty (such as 95% confidence interval) for any estimates. If relevant, these results should be by randomised group | **5-12** |
| **Ancillary analyses** | **18** | Results of any other analyses performed that could be used to inform the future definitive trial | **N/A** |
| **Harms** | **19** | All important harms or unintended effects in each group (for specific guidance see CONSORT for harms) | **10-12** |
|  | **19a** | If relevant, other important unintended consequences | **N/A** |
| **Discussion** | | | |
| **Limitations** | **20** | Pilot trial limitations, addressing sources of potential bias and remaining uncertainty about feasibility | **13** |
| **Generalisability** | **21** | Generalisability (applicability) of pilot trial methods and findings to future definitive trial and other studies | **12-13 Table 1** |
| **Interpretation** | **22** | Interpretation consistent with pilot trial objectives and findings, balancing potential benefits and harms, and considering other relevant evidence | **12-13** |
|  | **22a** | Implications for progression from pilot to future definitive trial, including any proposed amendments | **12-13, Table 1** |
| **Other information** | | |  |
| **Registration** | **23** | Registration number for pilot trial and name of trial registry | **N/A** |
| **Protocol** | **24** | Where the pilot trial protocol can be accessed, if available | **N/A** |
| **Funding** | **25** | Sources of funding and other support (such as supply of drugs), role of funders | **13** |
|  | **26** | Ethical approval or approval by research review committee, confirmed with reference number | **13** |
