# Supplementary material for: Feasibility of LifeLab Dublin: a co-designed health literacy intervention for socioeconomic disadvantaged schools
Source: Health Promot Int. 2026 Aug 18;41(4):daag127. doi: 10.1093/heapro/daag127 (PMC13483984; doi:10.1093/heapro/daag127)
Supplement: Supplementary file 1 [file daag127_supplementary_data.zip › Supplementaryfile2-Updated.docx]

**Supplementary file 2- Semi-structured interview and focus group question guides**

**Teacher Question Guide (Middle of intervention)**

1. How did you find the first three lessons plans?

- How effective do you feel they were?
- What are the strengths of it?
- What are the weaknesses?
- Would you change the order of the lessons?
- How was the content load?
- Were they easy to understand?

2. What was your honest opinion on the powerpoints and resources provided?

- How effective do you feel they were?
- What are the strengths of it?
- What are the weaknesses?
- What do you feel the students engaged with most?
- What was least engaging for them?

3. Do you think the LifeLab project aligns with the Wellbeing Curriculum?

- If so, where do you think it fits best/falls into the Social, Personal and Health Education/Wellbeing Curriculum?

4. In terms of the Social, Personal and Health Education/ Wellbeing curriculum how relevant are these lessons?

- What are the strengths of it?
- What are the weaknesses?
- Are there any topics discussed that you find irrelevant?
- Are there any topics you feel need to be discussed?
- Do you think the topics are discussed in enough detail?
- Do you think that three lessons was sufficient
- How do we make this as easy as possible for teachers to deliver/facilitate?
- What would you want us as researchers to provide to make your life easier?

Just to finish up, are there any other comments that you would like to make about your experience of Life Lab so far?

**Teacher Question Guide (End of intervention)**

1. How did you find the programme?

- How effective do you feel it was?
- What are the strengths of it?
- What are the weaknesses?
- Would you change the order of the lessons?
- How was the content load?
- Were they easy to understand?

2. What was your honest opinion on the photovoice project?

- How effective do you feel it was?
- What are the strengths of it?
- What are the weaknesses?
- Was it engaging for the students?
- What did you think about the guidelines?
- What is needed to improve the delivery of this project?

3. In terms of the SPHE/ Wellbeing curriculum how relevant is LifeLab?

- What are the strengths of it?
- What are the weaknesses?
- Are there any topics discussed that you find irrelevant?
- Are there any topics you feel need to be discussed?
- Do you think the topics are discussed in enough detail?
- Do you think that seven lessons was sufficient
- How do we make this as easy as possible for teachers to deliver/facilitate?
- What would you want us as researchers to provide to make your life easier?

4. Just to finish up, are there any other comments that you would like to make about your experience of Life Lab?

**Student question guide:**

1. What did you think of the LifeLab lessons you did in school?

- What did you enjoy about them?
- What did you not like about them?
- Were they interesting/ relevant?

2. What did you do in your photovoice project? (if they are not sure what this was, prompt them)

- Take pictures?
- What did you take pictures of? Why? Happy with what they could take pictures of?
- Carry out the activity in groups / on their own?
- Pictures on phones? School iPads?
- What kind of discussions did you have afterwards?

3. Did you like the photovoice project?

- What did you enjoy?
- What was less enjoyable?
- How did you find taking the pictures?
- How could we make it more enjoyable?

4. Did you learn anything from the photovoice project? / Did you learn anything from taking pictures and discussing them?

- What did you learn?
- Why do you think they learned X?
- Why did you feel it was not educational?
- How could we make it more educational?
- Did photovoice help you to learn about LifeLab? Why / why not?
- Did it help you to reflect on what you learned about in LifeLab?

5. If you were to do the photovoice project again, what would you change?

- How could we make it more interesting? More fun?
- How could we improve the not-so-good parts of it? Bring in the specific not-so-good parts that may have been brought up already.

6. Do you feel LifeLab will help you and your classmates’ understand health a little

more?

7. Do you think that there is anything else you would like to learn about in LifeLab?
