# Supplementary material for: Feasibility of LifeLab Dublin: a co-designed health literacy intervention for socioeconomic disadvantaged schools
Source: Health Promot Int. 2026 Aug 18;41(4):daag127. doi: 10.1093/heapro/daag127 (PMC13483984; doi:10.1093/heapro/daag127)
Supplement: Supplementary file 1 [file daag127_supplementary_data.zip › Supplementaryfile3-Updated.docx]

**Supplementary file 3- Teacher questionnaire**

| **Q no.** | **Question** | **Response options** | | | | | | |
| --- | --- | --- | --- | --- | --- | --- | --- | --- |
| **1** | **Did you complete all of the LifeLab lessons with your class?** | Yes | | | No | | | |
| **2** | **If no, which lessons did you not complete?*** | Lesson 1: Introduction to LifeLab | Lesson 2: LifeLab Health Topics | Lesson 3: LifeLab Characters | Lesson 4: Trial Project | Lesson 5: LifeLab Project | Lesson 6: LifeLab Project | Lesson 7: Reflection Lesson |
| **3** | **How relevant are the LifeLab lessons to your curriculum?** | Not relevant at all | Slightly relevant | Moderately relevant | | Very relevant | Extremely relevant | |
| **4** | **How engaging were the LifeLab lessons for students?** | Not engaging | Somewhat engaging | Moderately engaging | | Very engaging | Extremely engaging | |
| **5** | **How relevant are the LifeLab Health Topics to students?** | Not relevant at all | Slightly relevant | Moderately relevant | | Very relevant | Extremely relevant | |
| **6** | **Are the LifeLab Health Topics discussed in enough detail in the lessons?** | Yes | | | No | | | |
| **7** | **Are there any topics missing from the LifeLab lessons? If so please state below:** | Open-ended response | | | | | | |
| **8** | **How easy was it for you to deliver the LifeLab lessons?** | Very easy | Easy | Moderate | | Hard | Very hard | |
| **9** | **How would you evaluate the lesson plans?** | Bad | Fair | Good | | Very good | Excellent | |
| **10** | **Do you have any suggestions or ideas for how we could improve the lesson plans?** | Open-ended response | | | | | | |
| **11** | **How would you evaluate the scheme of work?** | Bad | Fair | Good | | Very good | Excellent | |
| **12** | **Do you have any suggestions or ideas for how we could improve the scheme of work?** | Open-ended response | | | | | | |
| **13** | **How would you evaluate the powerpoints?** | Bad | Fair | Good | | Very good | Excellent | |
| **14** | **Do you have any suggestions or ideas for how we could improve the powerpoints?** | Open-ended response | | | | | | |
| **15** | **Did any lesson stand out to you? (good or bad)*** | Lesson 1: Introduction to LifeLab | Lesson 2: The LifeLab Health Topics | Lesson 3: The LifeLab Characters | Lesson 4: The Trial Project | Lesson 5: The LifeLab Project | Lesson 6: The LifeLab Project | Lesson 7: The Reflection Lesson |
| **16** | **Can you explain your selection above?** | Open-ended response | | | | | | |
| **17** | **How would you evaluate the LifeLab project?** | Bad | Fair | Good | Very good | | Excellent | |
| **18** | **How engaging was the LifeLab project for students?** | Not engaging | Somewhat engaging | Moderately engaging | Very engaging | | Extremely engaging | |
| **19** | **How would you evaluate the delivering the LifeLab project?** | Very easy | Easy | Moderate | Hard | | Very hard | |
| **20** | **Would you use this style of project again?** | Yes | | | No | | | |
| **21** | **How would you evaluate the first visit to LifeLab in DCU?** | Bad | Fair | Good | Very good | | Excellent | |
| **22** | **How would you evaluate the second visit to LifeLab in DCU?** | Bad | Fair | Good | Very good | | Excellent | |
| **23** | **Is there anything that we could do to improve your visit to LifeLab in DCU?** | Open-ended response | | | | | | |
| **24** | **Would you recommend LifeLab to other teachers?** | Yes | | | No | | Maybe | |
| **25** | **In your opinion, what makes LifeLab different to other school initiatives?** | Open-ended response | | | | | | |
| **26** | **Do you have any further comments on the LifeLab programme that you'd like to add?** | Open-ended response | | | | | | |

*Multiple choice response option
