# Supplementary material for: Feasibility of LifeLab Dublin: a co-designed health literacy intervention for socioeconomic disadvantaged schools
Source: Health Promot Int. 2026 Aug 18;41(4):daag127. doi: 10.1093/heapro/daag127 (PMC13483984; doi:10.1093/heapro/daag127)
Supplement: Supplementary file 1 [file daag127_supplementary_data.zip › Supplementaryfile4-Updated.docx]

**Supplementary file 4- Data completeness and missing data**

|  | **Total N** | **% of total consented** | **Missing N** | **% missing of total consented** |
| --- | --- | --- | --- | --- |
| **Participants Invited** | 391 | - | - | - |
| **Consent provided** | 356 | - | - | - |
| **Baseline survey responses** | 306 | 86 | 50 | 14 |
| **Post-intervention survey responses** | 307 | 86.2 | 49 | 13.8 |
| **Follow-up survey responses** | 297 | 83.4 | 59 | 16.6 |
| **Survey responses at all 3 timepoints** | 242 | 68 | - | - |
| **Pre+ Post-intervention survey responses** | 277 | 77.8 | - | - |
| **Pre-intervention + follow-up survey responses** | 276 | 77.5 | - | - |

**-** not applicable

| **Frequency and percentage of the total responses excluded due to missing data in the scale** | | | | | | |
| --- | --- | --- | --- | --- | --- | --- |
|  | **Baseline** | | **Post-intervention** | | **Follow-up** | |
|  | **N** | **% (N= 309)** | **N** | **% (N= 307)** | **N** | **% (N= 298)** |
| Health Literacy score | 8 | 2.6 | 1 | 0.3 | 0 | 0.0 |
| WHO-5 Wellbeing score | 2 | 0.7 | 0 | 0.0 | 1 | 0.3 |
| Kidscreen- physical health score | 7 | 2.3 | 4 | 1.3 | 3 | 1.0 |
| Kidscreen- psychological wellbeing score | 3 | 1.0 | 1 | 0.3 | 1 | 0.3 |
| Kidscreen- parental support and autonomy score | 9 | 2.9 | 5 | 1.6 | 0 | 0.0 |
| Kidscreen- peer support score | 2 | 0.7 | 1 | 0.3 | 0 | 0.0 |
| Kidscreen- school learning and enjoyment score | 4 | 1.3 | 0 | 0.0 | 0 | 0.0 |
