# Supplementary material for: Feasibility of LifeLab Dublin: a co-designed health literacy intervention for socioeconomic disadvantaged schools
Source: Health Promot Int. 2026 Aug 18;41(4):daag127. doi: 10.1093/heapro/daag127 (PMC13483984; doi:10.1093/heapro/daag127)
Supplement: Supplementary file 1 [file daag127_supplementary_data.zip › Supplementaryfile5-Updated.docx]

**Supplementary file 5- Frequently asked questions from the student survey**

| **Scale** | **Item** | **Rephrased as..*** |
| --- | --- | --- |
| Health Literacy in School Aged Children survey | Item 3 ‘I can compare health related information from different sources’ | Could you take health information from online and from your doctor (or in school) and compare it? |
|  | Item 9 ‘I can usually figure out if some health information is right or wrong’ | Can you tell if what you are seeing online is right or wrong, true or false? |
|  | Item 10 ‘I can give reasons for choices I make regarding health’ | Could you explain why you do certain things regarding your health? |
| WHO-5 Wellbeing index | Item 3 ‘I have felt active and vigorous’ | full of energy |
| Kidscreen-27 survey | response option ‘seldom’ | Not often |
|  | Item 18 ‘have you had enough money to do the same things as your friends’ | Do you ever have to say no to doing things with friends because of money? |
|  | Item 19 ‘have you had enough money for your expenses’ | In general, do you have to worry about money? |
|  | Item 25 ‘have you got on well at school’ | In general, how is your school experience? |

*The survey was administered as per recommended protocols however, if students asked for clarification during survey administration these phrases were used by the research team to provide clarity
